# Response of recalcitrant Blaschkoid psoriasis to bimekizumab: A case report

**DOI:** 10.1016/j.jdcr.2025.10.078

**Published:** 2025-12-04

**Authors:** Hannah Verma, Marguerite Meariman, Benjamin Gerstein, Kateryna Karpoff, Raphaella Lambert, Grace Rabinowitz, Jonas Adalsteinsson, George Niedt, Reena Rupani, Annemarie Uliasz, Nicholas Gulati

**Affiliations:** Kimberly and Eric J. Waldman Department of Dermatology, Icahn School of Medicine at Mount Sinai, New York, New York

**Keywords:** bimekizumab, biologics, Blaschkoid psoriasis, ILVEN, linear psoriasis

## Introduction

Blaschkoid psoriasis, also referred to as linear psoriasis, is a rare subtype of psoriasis that presents as plaques along the lines of Blaschko, which represent developmental pathways of epidermal cell differentiation and migration.^1^ These lines do not follow the arrangements of vasculature or dermatomes.^2^ Its pathogenesis is thought to be a product of mosaicism.^3^ Blaschkoid psoriasis is often subject to delayed diagnosis and trials of multiple systemic treatments, with limited data on treatment efficacy.^3^ It can easily be confused with inflammatory linear verrucous epidermal nevus (ILVEN), a clinical mimicker of Blaschkoid psoriasis that often arises in early childhood (typically before age 5), with an estimated prevalence of 1 in 1000 births.^4^ Here, we present a case of an adult male with recalcitrant Blaschkoid psoriasis who demonstrated improvement with bimekizumab.

## Case report

A 32-year-old male with hypertension presented to dermatology clinic for evaluation and management of linear, erythematous, scaly plaques along the lines of Blaschko involving his back, right arm, and right inner and outer thigh, present for over 23 years. He also presented with a discrete plaque with silver scale on his left elbow. The patient reported a family history of psoriasis in both his paternal and maternal grandmothers. He denied joint pain, was a nonsmoker, and lacked any joint, scalp, or nail involvement. He was presumed to have ILVEN, and was treated with excimer laser, high potency topical steroids, methotrexate (mild improvement with recurrence), etanercept (no improvement), adalimumab (discontinued after 3 months), and ustekinumab (no improvement). The lesions were surgically excised and treated with CO_2_ laser, but later recurred.

**Fig 1:**
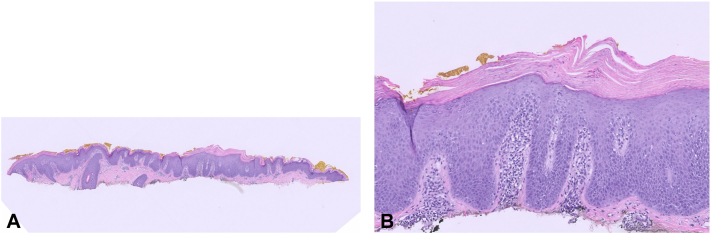
**A,** On low power, there is psoriasiform hyperplasia of the epidermis with overlying parakeratosis. Areas of alternating para- and orthokeratosis were present in some sections. **B,** Collections of neutrophils were present in the horn in some foci, with surrounding parakeratosis and overlying diffuse hypogranulosis.

A biopsy in 2021 demonstrated psoriasiform dermatitis. Microscopic evaluation of the tissue was notable for psoriasiform hyperplasia of the epidermis, with collections of neutrophils in some foci, as well as areas of alternating para- and orthokeratosis (Fig 1, *A* and *B*).

**Fig 2:**
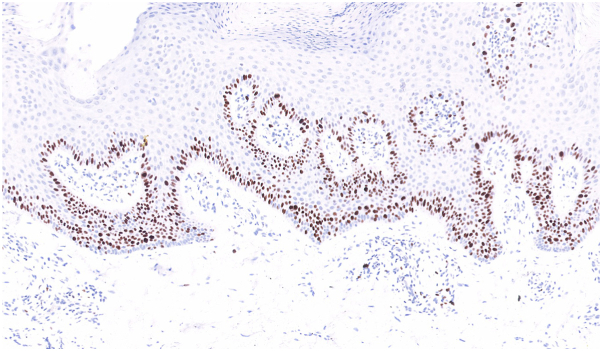
Moderate to elevated Ki-67 staining was present in the epidermis.

Additional staining with Ki-67, a commonly used marker of cell proliferation in dermatology, was performed, revealing elevated levels of Ki-67, favoring a diagnosis of Blaschkoid psoriasis over ILVEN (Fig 2).

**Fig 3:**
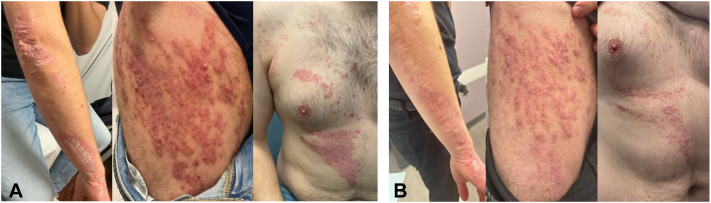
Clinical photographs of Blaschkoid psoriasis before **(A)** and after 10 weeks of treatment with bimekizumab **(B)**.

Subsequently, the patient failed treatment with calcipotriene 0.005% ointment, tapinarof 1% cream, deucravacitinib, and apremilast in concert with halobetasol. At his visit in October 2024, the body surface area coverage of his psoriasis was estimated to be 20% and the Investigator’s Global Assessment score of severity was 4 (Fig 3, *A*). Based on the patient’s severity of disease, as well as lack of response to multiple other systemic agents with different mechanisms of action, treatment with bimekizumab was initiated at the standard dose of 320 mg every 4 weeks for the first 16 weeks, followed by every 8 weeks thereafter. On the 10th week of treatment, the patient returned to clinic with significantly improved erythema, scaling, and flattening of plaques, with no adverse events, and continued to use halobetasol on his knuckles (body surface area 15%, Investigator’s Global Assessment 3) (Fig 3, *B*). At his 6-month follow-up, the patient demonstrated continued improvement with further flattening of plaques and reduction of erythema, and he was able to discontinue halobetasol (body surface area 15%, Investigator’s Global Assessment 2); the patient reported he was pleased with this improvement.

## Discussion

Clinical differentiation of Blaschkoid psoriasis and ILVEN can be challenging, as both present with psoriasiform lesions along the lines of Blaschko. There is considerable debate regarding whether the conditions overlap or share similar cellular mutations.^3^ Generally, ILVEN presents at an earlier age, progresses more slowly, is less responsive to treatment, and exhibits increased pruritus compared to Blaschkoid psoriasis.^3^ Blaschkoid psoriasis is rare, with no estimated prevalence. It most commonly presents in early adulthood, shows no consistent sex or racial predilection, and is frequently subject to diagnostic delay.^5^ It is more treatment-resistant than psoriasis vulgaris, with many patients requiring systemic or biologic therapy.^5^ Histologically, ILVEN typically demonstrates parakeratosis and orthokeratosis with a reduced granular layer, while Blaschkoid psoriasis more commonly shows continuous parakeratosis, micro-abscesses and absence of the granular layer, and neutrophilic infiltration.^3^ Nonetheless, overlapping clinical and histopathological features blur the distinction between these entities. Immunohistochemical staining can be useful in unresolved cases; increased expression of involucrin and Ki-67 is more commonly seen in Blaschkoid psoriasis, while ILVEN demonstrates higher keratin-10 staining.^1^^,^^6^ More specifically, the presence of moderate to elevated Ki-67 staining in the epidermis can be used to favor psoriasis.^6^^,^^7^

Blaschkoid psoriasis may present concurrently with or superimposed upon psoriasis vulgaris, as in this patient, which is classified as type II Blaschkoid linear psoriasis.^5^ Type II disease is more likely to be treatment resistant and require biologic therapy compared to those with type I, which is Blaschkoid psoriasis without coexisting classical psoriasis lesions.^5^ This patient’s partial clinical response supports the diagnosis of Blaschkoid psoriasis and correlates with the elevated Ki-67 staining.

Given the treatment-resistant nature of this patient’s condition to biologics with other mechanisms of action, bimekizumab was selected. Bimekizumab is currently Food and Drug Administration-approved for moderate-to-severe plaque psoriasis in candidates for systemic therapy. Its mechanism of action involves dual inhibition of interleukin (IL)-17A and IL-17F, which are implicated in IL-23-dependent and IL-23-independent psoriasis. In case studies of 2 pediatric patients with Blaschkoid psoriasis, ixekizumab (an IL-17A inhibitor) and secukinumab (an IL-17 inhibitor) treatment produced near-complete resolution of lesions.^2^^,^^8^ The authors of the latter study suggested that IL-17 inhibition can be effective for Blaschkoid psoriasis lesions with inflammatory, hyperkeratotic features.^2^ Enrichment analysis shows Blaschkoid psoriasis has elevated IL-17 associated activity compared to controls, and that there are different immunophenotypic profiles between Blaschkoid psoriasis and psoriasis vulgaris.^9^ Bimekizumab, with additional inhibition of the IL-17F isomer, may be a favorable treatment option. Similarly, 2 biologic-naive adults with linear psoriasis reported significant improvement after administration of ixekizumab.^10^ In this case, our adult patient had tried multiple biologics, including TNF-alpha, IL-23, and TYK2 inhibitors, representing a unique case of biologic-resistant Blaschkoid psoriasis that improved with IL-17A and IL-17F inhibition. Compared to psoriasis vulgaris, and pediatric or biologic-naïve adult cases of Blaschkoid psoriasis treated with IL-17 inhibitors, treatment response in this case was attenuated, with partial clearance after 6 months.^2^^,^^8^^,^^10^ While data on systemic therapy for treatment-resistant Blaschkoid psoriasis remains limited, this case highlights the potential utility of bimekizumab.

## Conflicts of interest

Dr Gulati is an employee of Mount Sinai and has served as a consultant for Almirall, Daiichi Sankyo, and Primus Pharmaceuticals. Drs Meariman, Adalsteinsson, Niedt, Rupani, Uliasz, and Authors Verma, Gerstein, Karpoff, Lambert, and Rabinowitz have no conflicts of interest to declare.
